# A study on the chemical stability of cholesterol-lowering drugs in concomitant simple suspensions with magnesium oxide

**DOI:** 10.1186/s40780-023-00301-1

**Published:** 2023-08-29

**Authors:** Ginjiro Kato, Hidemichi Mitome, Yusura Miyauchi, Syu Takeda, Yoshito Toyota, Noriaki Hidaka, Mamoru Tanaka, Kazuki Akira

**Affiliations:** 1 Laboratory of Pharmaceutical Analytical Chemistry, College of Pharmaceutical Sciences, Matsuyama University, 4-2 Bunkyo-Cho, Matsuyama, Ehime 790-8578 Japan; 2 Division of Pharmacy, Ehime University Hospital, 454 Shitsukawa, Toon, Ehime 791-0295 Japan

**Keywords:** Simple suspension method, Dysphagia, Cholesterol-lowering drug, Magnesium oxide, Stability, Degradation

## Abstract

**Background:**

Difficulty in taking solid medicines is a common issue particularly for the elderly because of a decline in swallowing function, also known as dysphagia. For patients with such a dysfunction, a simple suspension method, in which solid medicines are disintegrated and suspended using warm water, has been developed and widely used in Japanese clinical settings. However, there is little information on drug stability in the simple co-suspension of multiple formulations especially including acidic or alkaline ones. In this study, the chemical stability of typical cholesterol-lowering drugs was investigated in a simple co-suspension with alkaline magnesium oxide (MgO) which is frequently used as a laxative or antacid in Japan.

**Methods:**

A cholesterol-lowering drug (one tablet) was soaked with or without MgO in warm water (55°C), and the vessel was left at room temperature for 10 min or 5 h. The suspensions prepared were then analyzed by high-performance liquid chromatography. Degradation products were analyzed by nuclear magnetic resonance spectroscopy and mass spectrometry for the structural elucidation.

**Results:**

In the simple co-suspension with MgO, no significant degradation was observed for atorvastatin or pravastatin, while a significant decrease of the recovery from the co-suspension was observed for rosuvastatin after 5 h. On the other hand, simvastatin and ezetimibe co-suspended with MgO were partially degraded to simvastatin acid and a pyran compound, respectively.

**Conclusions:**

A simple co-suspension with MgO is feasible for atorvastatin, pravastatin, and rosuvastatin, although the rosuvastatin tablet should not be left soaking for a long time. Further it is inadvisable to suspend simvastatin or ezetimibe together with MgO because of their partial degradation.

**Supplementary Information:**

The online version contains supplementary material available at 10.1186/s40780-023-00301-1.

## Background

A decline in swallowing function is a common issue in the elderly population and it makes it difficult for some patients to ingest solid oral medicines as well as food [1, 2]. This swallowing dysfunction, so-called dysphagia, can also occur in patients with various acute and chronic diseases such as stroke, depression, dementia, and structural or functional abnormalities of the oropharynx. When solid oral medicines are inapplicable for the patients with swallowing dysfunction, other suitable formulations or alternative routes of administration are required. However, such medications are not available in most cases. Thus, it is customary practice to prepare aqueous suspensions of tablets and capsules followed by tube administration [3, 4].

The suspensions have been commonly prepared by a crushing method where tablets are pulverized and capsules are opened. However, there have been reports of issues with the method such as loss of drugs or exposure of clinical staff to drugs [5–7]. Thus, a simple suspension method (SSM) has been developed in order to solve these issues [8]. In the SSM, tablets and capsules are soaked in warm water (55°C) and the vessels are left at room temperature for 10 min, by which the formulations are disintegrated and easily suspended after mixing. The suspension formation is facilitated in the warm water, which reduces the risk of clogging feeding tubes. The feasibility of SSM has been extensively tested with respect to ease of suspension and passage through feeding tubes, and found to be applicable to most solid oral medicines [8–11]. The method is simple and convenient and thus widely used in Japanese clinical settings. However, concerns about the chemical stability of drugs in the SSM have not been eliminated [8]. In particular, little is known about the chemical incompatibility in the concomitant simple suspensions (hereafter simply called ‘co-suspension’) of plural medicines. For example, some drugs may degrade when they are subjected to the SSM with acidic or alkaline drugs. This is an important point because elderly patients usually ingest many kinds of drugs at the same time.

Magnesium oxide (MgO) has been widely used as a laxative as well as an antacid in East Asia [12]. The use of the drug has become more frequent due to an increase in the number of elderly patients with chronic constipation in an aging society [13]. Thus, MgO is often used together with other drugs related to life-style diseases as well as common diseases. MgO is slightly soluble in water to show basicity, and magnesium ions are known to show some interactions such as complex formation with various drugs [14]. Thus, it is necessary to evaluate the chemical stability of drugs concomitantly suspended with MgO under an SSM condition. Aoki et al. [15] reported that no degradation occurred in the co-suspension of MgO and clopidogrel bisulfate, which has an ester moiety. On the contrary, Suryani et al. [16] reported that when ester prodrugs, acemetacin and cefpodoxime proxetil, were subjected to the SSM with MgO, the ester bonds were hydrolyzed. These chemical changes were considered to be serious because of the potential occurrence of gastrointestinal injury or some reduction of drug efficacy.

Cholesterol-lowering drugs (CLDs) such as statins and ezetimibe are most effective for the treatment of dyslipidemia, which is one of the major risk factors for cardiovascular disease [17, 18]. This disease is particularly prevalent in the elderly. Due to the large number of dyslipidemic patients, use of the drugs has become more frequent, which increases the probability of the simultaneous prescription with MgO. Most CLDs possess partial structures, which are susceptible to chemical changes. Their chemical stability under alkaline conditions has been previously investigated [19–28]. However, the situation is different under an SSM condition with MgO because the drugs are not always fully soluble under the condition, and various additives contained in their formulations may influence chemical stability. Thus, in this paper, the chemical stability of typical CLDs concomitantly suspended with MgO under an SSM condition was investigated.

## Methods

### Reagents

The following formulations of CLDs were used: Atorvastatin tablets 5 mg (Sawai, Osaka, Japan), Pravastatin sodium tablets 5 mg (Sawai, Osaka, Japan), Rosuvastatin tablets 2.5 mg (DSEP, Tokyo, Japan), Simvastatin tablets 5 mg (Teva Takeda Yakuhin, Aichi, Japan), Ezetimibe tablets 10 mg (DSEP, Tokyo, Japan). Magmitt tablets 330 mg (Nihon Shinyaku, Kyoto, Japan) were used as an MgO containing formulation. The tablets of atorvastatin, pravastatin, rosuvastatin, simvastatin, ezetimibe, and MgO are hereafter referred to as AS, PS, RS, SS, EZ, and MG, respectively. Atorvastatin calcium hydrate (99.8%), simvastatin (100%), and ezetimibe (96.3%) were purchased from Tokyo Chemical Industry (Tokyo, Japan). Pravastatin sodium (99.9%), rosuvastatin calcium (99.7%), distilled water, acetonitrile, methanol, trifluoroacetic acid, and sodium dihydrogen phosphate dihydrate were purchased from Fujifilm Wako Chemicals (Osaka, Japan). Distilled water, acetonitrile, and methanol were of high-performance liquid chromatography (HPLC) grade. An aqueous solution of phosphoric acid (0.5 M) and chloroform were purchased from Kanto Chemical (Tokyo, Japan). Weakly acidic cation exchange resin AMBERLITE® (IRC76) was purchased from ORGANO (Tokyo, Japan). A membrane filter DISMIC®—03 JP (0.50 μm) was purchased from Toyo Roshi Kaisha (Tokyo, Japan). Deuterated chloroform (CDCl_3_) containing 0.05% tetramethylsilane was purchased from Eurisotop (Massachusetts, USA). Deuterated dimethyl sulfoxide (DMSO-*d*_6_) containing 0.03% tetramethylsilane was purchased from Acros Organics (Massachusetts, USA).

### Preparation of simple suspensions

One CLD tablet was placed with or without MG (one tablet) in a 50-mL plastic centrifuge tube containing 20 mL of warm distilled water (55°C). The centrifuge tube was allowed to stand at room temperature (*ca.* 25°C) for 10 min or 5 h. The mixture was then vortexed for 1 min to prepare a suspension. The subsequent treatments were immediately performed according to method I or II (see below). In separate experiments, the simple suspension prepared after 10 min soakage was centrifuged and the aqueous supernatant was directly subjected to HPLC analyses and pH measurements using InLab® Routine Pro (Mettler Toledo, Ohio, USA). The suspension preparation was performed three times for each CLD tablet in any of the experiments.

### Treatment of simple suspensions

The simple suspensions were treated as follows, and the resulting solutions were analyzed by HPLC.

Method I: The simple suspension was completely transferred into a 100-mL volumetric flask, where the tube was washed by methanol (for AS, PS, RS, and EZ) or acetonitrile (for SS) and the washing solvent was put into the same flask. The mixture in the flask totaled 100 mL with the respective organic solvents, followed by sonication for 1 min unless otherwise noted.

Method II: For SS and EZ, the simple suspension was centrifuged, and the aqueous supernatant (18 mL) was neutralized by passing it through a column of the cation exchange resin. Eighteen milliliters of acetonitrile for SS or methanol for EZ were added to the residue of centrifugation, and the mixture was vortexed for 1 min and centrifuged again. The supernatant was also passed through the same cation exchange column. The same treatment was conducted again for the residue of the centrifugation. The effluents from the column were collected and totaled 250 mL by the respective organic solvents.

### HPLC analysis

HPLC analyses were performed using a Shimadzu HPLC system (Kyoto, Japan) as described in our previous paper [29]. The reversed-phase HPLC analyses were performed modifying the conditions in the literature [30]. An Atlantis T3 column (4.6 × 250 mm, 5 μm; Waters, Milford, USA) fitted with a guard column (4.6 × 20 mm, 5 μm) was used. The mobile phases and detection wavelengths used are summarized in Table 1. The HPLC system was operated isocratically at a flow rate of 1.0 mL/min at 25°C. The samples were appropriately diluted and injected after being filtered using the membrane filter. The injection volume of the sample was 10 μL. Chromatograms were obtained within 30 min after the suspension preparation.

**Table 1:** Reversed-phase HPLC conditions

Formulation	Composition of mobile phase^a^ (buffer / CH_3_CN)	Wavelength (nm)
AS	35 / 65	245
PS	60 / 40	234
RS	45 / 55	242
SS	15 / 85	238
EZ	40 / 60	232

^a^Twenty millimolar phosphoric acid-sodium dihydrogen phosphate buffer (pH 2.5) was used

### Validation method of HPLC analysis

Standard stock solutions of atorvastatin calcium hydrate, pravastatin sodium, rosuvastatin calcium, simvastatin, and ezetimibe were prepared in methanol (*ca*. 100 μg/mL). The solutions for the validation were prepared from stock solutions. The calibration curves were constructed by triplicate analysis of the five concentrations of the samples, whose ranges were one fourth or one eighth to twice the expected drug concentrations in the diluted samples for the HPLC analyses. The expected drug concentrations for atorvastatin, pravastatin, rosuvastatin, simvastatin, and ezetimibe were 2.0, 2.0, 1.0, 2.0, and 4.0 μg/mL, respectively. Quality control samples were prepared at three concentrations (middle, high, low) within the ranges of the calibration curves. The three concentrations were half of the expected drug concentrations, the three-fold concentrations, and 40 to 70% less concentrations. The precision and repeatability of the HPLC methods were determined by intra- and inter-day variations through repeated analyses (*N* = 5) of quality control samples on three different days. Various diluted samples were analyzed five times, and the limit of detection (LOD) and limit of quantitation (LOQ) were determined based on the concentrations with signal to noise ratios of three and 10, respectively.

### Structural elucidation of degradation products

The degradation product from the SS was extracted with chloroform from the aqueous supernatants of the co-suspensions of SS and MG for structural analysis using nuclear magnetic resonance (NMR) spectroscopy. ^1^H and ^1^H-decoupled ^13^C NMR spectra and two-dimensional NMR spectra were measured with an AVANCE 500 NMR spectrometer (11.75 T, Bruker Japan, Kanagawa, Japan) using 5-mm sample tubes at 300 K. The aqueous supernatants of the co-suspensions of SS and MG were also injected into the HPLC apparatus in order to collect the fractions containing the degradation product. The HPLC conditions were the same as described above, except the mobile phase was a mixture of 0.1% trifluoroacetic acid and acetonitrile (15/85). The fractions were directly analyzed with a micro TOF-Q spectrometer (Bruker Daltonics, Kanagawa, Japan) in order to obtain the high-resolution electrospray ionization mass spectrum (ESI–MS).

The aqueous methanolic solution, which was obtained from the co-suspensions of EZ and MG, was injected into the HPLC apparatus to isolate the degradation product from EZ. The HPLC conditions were the same as described above, except the mobile phase was a mixture of 0.1% trifluoroacetic acid and acetonitrile (40/60). The NMR spectra and ESI–MS of the isolated degradation product were measured using the above spectrometers.

### Statistical analysis

All data are expressed as the mean ± standard deviation of at least three experiments. All statistical analyses were performed using one-way ANOVA followed by Tukey’s test to determine significance. Statistical significance was set at *p* < 0.05.

## Results

### Preparation of suspensions

**Fig. 1:**
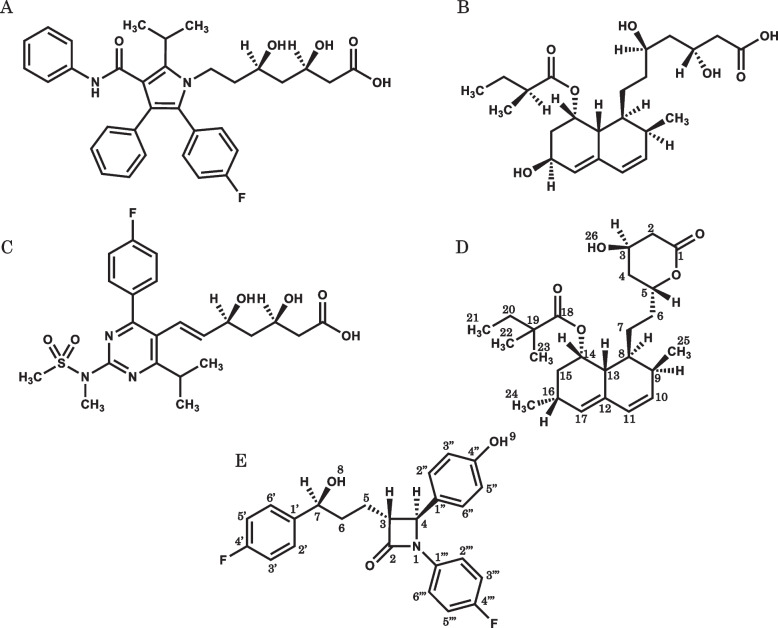
Chemical structures of CLDs examined. symbols: **A** atorvastatin; **B** pravastatin; **C** rosuvastatin; **D** simvastatin; **E** ezetimibe

The CLDs examined in this study are shown in Fig. 1. These drugs have partial structures such as ester bonds, which are labile to the alkaline conditions, or coordination groups such as carboxy groups. The SSM was applied to their tablets, which were disintegrated with or without MG, and their suspensions successfully prepared. The pH values of the aqueous supernatants of the suspensions without MG were near neutral except for AS, whereas those of the co-suspensions with MG were fairly basic (Table 2).

**Table 2:** pH values of supernatants of the simple suspensions prepared after soak for 10 min^a^

Formulation	pH	
	Without MG	With MG
AS	9.5 ± 0.14	10.7 ± 0.04
PS	7.0 ± 0.21	10.8 ± 0.01
RS	6.5 ± 0.09	10.8 ± 0.06
SS	6.9 ± 0.24	10.7 ± 0.08
EZ	7.7 ± 0.41	10.8 ± 0.04

^a^Each formulation (one tablet) was subjected three times to the SSM with or without MG (one tablet). When only MG (one tablet) was subjected three times to the SSM, the pH value of the supernatant was 10.7 ± 0.10

### Stability studies

**Fig. 2:**
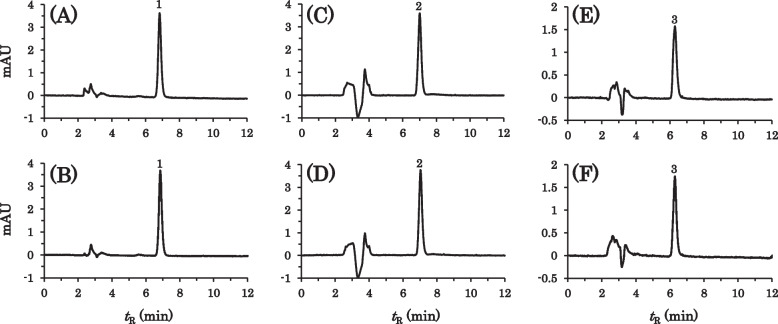
HPLC chromatograms of AS (left), PS (middle), and RS (right) suspended with or without MG under the SSM condition. The suspensions of the following formulations were prepared and treated by method I followed by HPLC analysis, immediately after disintegration for 10 min. The mobile phases and detection wavelengths; see Table 1. **A** AS; **B** AS + MG; **C** PS; **D** PS + MG; **E** RS; **F** RS + MG. symbols: 1, atorvastatin; 2, pravastatin; 3, rosuvastatin

**Fig. 3:**
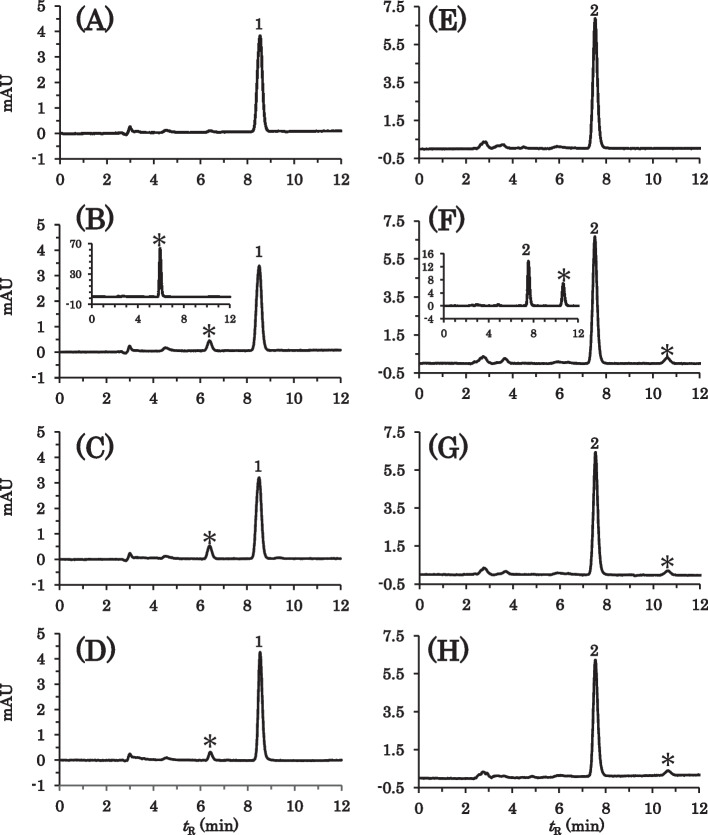
HPLC chromatograms of SS (left) and EZ (right) suspended with or without MG under the SSM condition. The suspensions of the following formulations were prepared and treated by method I (**A**, **B**, **C**, **E**, **F**, **G**) or method II (**D**, **H**) and the resulting solutions were analyzed by HPLC. Unless otherwise noted, the analyses were performed immediately after disintegration for 10 min. The insets in panels **B** and **F** show the chromatograms of the aqueous supernatants of the co-suspensions with MG. The mobile phases and detection wavelengths; see Table 1. **A** SS; **B** and **D** SS + MG; **C** SS + MG (5 h later); **E** EZ; **F** and **H** EZ + MG; **G** EZ + MG (5 h later). symbols: 1, simvastatin; 2, ezetimibe; *, degradation product

The simple suspensions were treated by method I and the extracts were analyzed by HPLC. For AS, PS, and RS, only the peaks of the medicinal ingredients were observed with or without MG at any wavelength, as shown in Fig. 2. For SS, one degradation peak was observed at *t*_R_ 6.4 min when co-suspended with MG, whereas the degradation peak was negligible without MG, as shown in Fig. 3A-C. The degradation product had a shorter retention time in the reversed-phase column, suggesting that simvastatin was transformed into a more hydrophilic product by hydrolysis of the ester bond or lactone moiety. For EZ, a minute degradation peak was observed at *t*_R_ 10.6 min when co-suspended with MG, whereas no degradation peak was observed without MG, as shown in Fig. 3E-G. The EZ degradation product with a longer retention time was considered to be more hydrophobic than ezetimibe. In order to eliminate the possibility of further degradation during the sample treatment by method I, the co-suspension of SS with MG or EZ with MG was analyzed after immediate neutralization by the cation exchange column (method II). Consequently, the degradation peaks were also observed to a similar degree, as shown Fig. 3D and H. In addition, these degradation peaks were also observed when the aqueous supernatants of the co-suspensions with MG were directly injected into the HPLC system, as shown in the insets of Fig. 3B and F. The absorption spectra of these degradation products were almost identical to those of the corresponding parent drugs, indicating that the chromophore structures remained unchanged.

The amounts of drugs in the extracts were measured based on the HPLC peak areas, and the percentages of the measured values to the labeled amounts of the tablets were calculated (Table 3). In the suspensions without MG, the percentages of the individual drugs were 98 to 105% after 10 min, and no significant decreases of the percentages were observed after 5 h. In the co-suspensions with MG, there were no significant decreases in the percentages of atorvastatin and pravastatin compared with the case without MG, while a considerable decrease of the percentage was observed for rosuvastatin after 5 h.

**Table 3:** Quantitative determination of the medicinal ingredients in the simple suspensions^a^

Formulation	Without MG (%)		With MG (%)	
	After 10 min	After 5 h	After 10 min	After 5 h
AS	99.9 ± 3.2	94.2 ± 0.9	96.3 ± 2.2	91.7 ± 4.6
PS	100.5 ± 2.7	101.9 ± 3.4	96.4 ± 3.1	94.3 ± 3.4
RS^b^	97.6 ± 1.4	92.8 ± 1.9	95.3 ± 0.7	68.9 ± 6.8^†^
SS	98.2 ± 0.9	96.4 ± 0.3	88.2 ± 1.0^*^	86.4 ± 2.1^†^
	(98.4 ± 1.9)	(97.7 ± 1.6)	(88.3 ± 2.1)^*^	(88.8 ± 2.5)^†^
EZ	104.7 ± 1.7	103.9 ± 2.3	98.6 ± 2.0^*^	99.9 ± 1.5
	(100.6 ± 1.0)	(99.6 ± 0.8)	(98.2 ± 2.3)	(92.9 ± 2.6)^†^

^*^p < 0.05 vs. after 10 min without MG

^†^*p* < 0.05 vs. after 5 h without MG

^a^The tablets were soaked into the warm water, suspended and treated by method I. The percentages of the quantitative values to the labeled amounts of the individual tablets were then calculated. For SS and EZ, the suspensions were also treated by method II, and the quantitative results are shown in the parentheses

^b^The mixtures in the 100-mL volumetric flasks were sonicated for 10 min

### Structural elucidation of degradation products

**Fig. 4:**
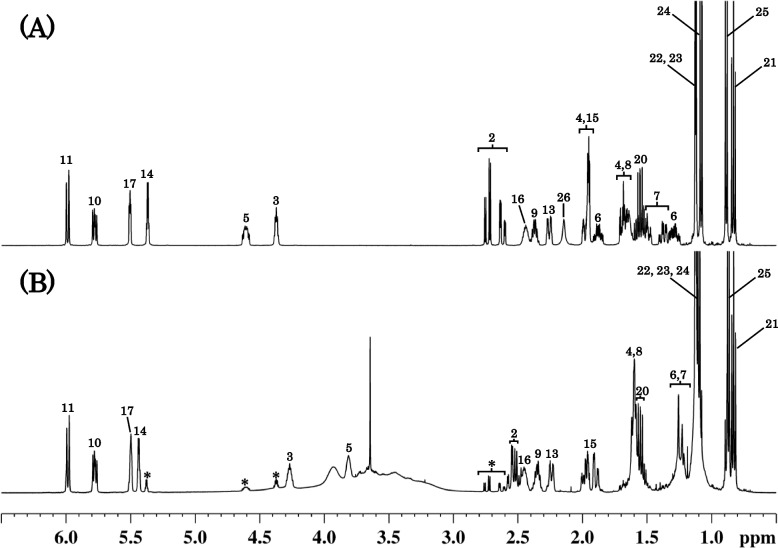
^1^H NMR spectra of simvastatin (**A**) and the chloroform extract from the co-suspension of SS and MG (**B**). The spectra were measured in CDCl_3_. The chloroform extract was found to mainly contain the degradation product, simvastatin acid, with a small amount of the unchanged simvastatin (with asterisk). The spectrum (**B**) was considered to also contain some signals due to the additives of formulations. The numeric characters on the spectra (**A**) and (**B**) show assignments of the signals to the positions of the chemical structures shown in Fig. 1 (simvastatin) and Fig. 5 (simvastatin acid), respectively

**Fig. 5:**
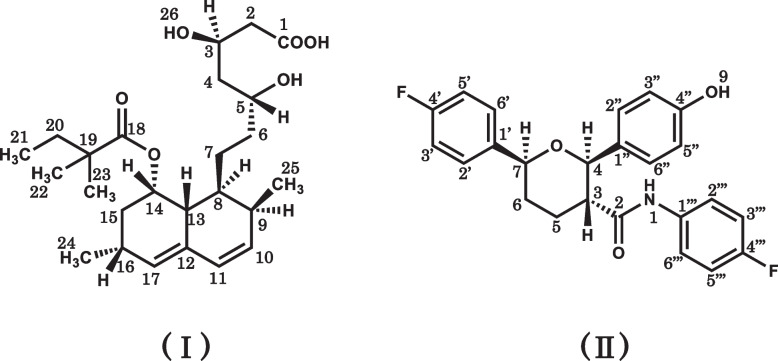
Chemical structures of the degradation products. I: simvastatin acid, II: (2*R*,3*R*,6*S*)-*N*,6-bis(4-fluorophenyl)-2-(4-hydroxyphenyl)-3,4,5,6-tetrahydro-2*H*-pyran-3-carboxamide

The chloroform extract from the co-suspension of SS with MG was confirmed to contain one major HPLC peak due to the degradation product. The ^1^H NMR spectra of simvastatin and the extract are shown in Fig. 4. In Fig. 4B, major signals due to the degradation product were observed with minor ones due to the unchanged simvastatin. The signal (H5), due to the lactone moiety of simvastatin, greatly shifted in the degradation product. The accurate mass spectrum of the degradation product showed an ion peak [M + Na]^+^ at *m/z* 459.2737, which showed that the molecular formula of the degradation product was C_25_H_40_O_6_. The molecular weight, 436, was 18 (water molecules) greater than that of simvastatin (C_25_H_38_O_5_). These data indicate the hydrolysis of the lactone moiety of simvastatin, resulting in the formation of simvastatin acid (Fig. 5) as previously reported in the literature [25]. Almost all the major ^1^H signals shown in Fig. 4B were assigned to the individual protons of simvastatin acid. Thus, the SS degradation product was identified as simvastatin acid, which was also supported by the ^13^C NMR and two-dimensional NMR spectroscopic data (Supplemental Figs. 1, 2, 3 and 4).

When the ^1^H NMR spectrum (Supplemental Fig. 5) of the degradation product from EZ was compared with that of ezetimibe, the ^1^H signals due to the 5- and 6-positions of ezetimibe (see Fig. 1) were found to be greatly changed in terms of the chemical shifts and signal shapes. The accurate mass spectrum of the degradation product showed an ion peak at *m/z* 432.1402 [M + Na]^+^, indicating that the molecular formula was C_24_H_21_F_2_NO_3_. These data showed that the product was the structural isomer of ezetimibe, a pyran compound (Fig. 5, compound II) as previously reported in the literature [27]. The ^1^H NMR spectrum of the product was substantially identical to the spectrum data of the pyran compound in the literature. The structure was supported by the ^13^C and two-dimensional NMR spectroscopic data (Supplemental Figs. 6, 7, 8 and 9).

### Validation of the HPLC method

The HPLC methods were validated for the individual drugs in terms of linearity, precision, LOD, and LOQ as shown in Supplemental Tables 1 and 2. The calibration curves exhibited good linearity in the tested concentrations ranges, with the coefficients of determination (*r*^2^) being greater than 0.999. The intra- and inter-day relative standard deviations were less than 1.9% and 2.4%, respectively. The LOD and LOQ were in the range of 16 ng/mL to 65 ng/mL and 47 ng/mL to 262 ng/mL, respectively. All of the validation parameters were within the acceptance ranges.

## Discussion

There have been many reports on the chemical stability under aqueous alkaline conditions for the CLDs examined in this study (Table 4). In most cases, relatively strong conditions were applied, and the drugs were more or less decomposed. Thus, the chemical stability for the individual drugs needed to be investigated under the condition of SSM with MG. In clinical settings, the prepared simple suspensions are occasionally left for long time before administration due to business reasons [31]. Thus, in this study, simple suspensions after being soaked for 5 h as well as 10 min were prepared and analyzed, assuming that the maximum time to be left was 5 h to be on the safe side.

**Table 4:** Degradation studies of cholesterol-lowering drugs under alkaline conditions

Drug	Condition	Degradation rate (%)	Literature
Atorvastatin	1 M NaOH, 60°C, 10 min	4	Khedr et al. [19]
Pravastatin	1 M NaOH, 80°C, 1 h	89	Önal et al. [21]
Rosuvastatin	0.01 M NaOH, 85°C, 10 h	0	Machairas et al. [22]
Simvastatin	28 mM phosphate buffer (pH 8), 60°C , 1.5 h	*ca*. 80^a^	Álvarez-Lueje et al. [24]
Ezetimibe	0.01 M, NaOH, 25°C, 5 min	24	Goel et al. [26]

^a^The value was estimated from the chromatogram in the literature

From the experimental results, it was shown that atorvastatin and pravastatin were chemically stable under the SSM condition with or without MG. In contrast, the recovery of rosuvastatin from the co-suspensions with MG considerably decreased after 5 h. The decrease seems to be due to adsorption of rosuvastatin to the large amounts of MgO and/or formation of undissolved complex with Mg^2+^ [14]. These putative interactions of rosuvastatin may, at least temporarily, remain unchanged in the stomach when the co-suspension is ingested after meals, because the acidity of the stomach contents considerably decreases (pH ~ 5) due to meals [32], and MgO neutralize the gastric acid. It was reported that the blood concentration of rosuvastatin was markedly decreased when the drug was ingested at the same time with magnesium hydroxide [33]. Phenomena similar to the above ones can be presumed to occur in the stomach, leading to the delayed and reduced absorption of rosuvastatin into the gastrointestinal tract. As shown in Table 3, the percentages of simvastatin to the labeled amount were significantly decreased by the co-suspension with MG regardless of the immersion time and neutralization. Although the percentages of ezetimibe were also decreased by the co-suspension with MG, the decrease was not necessarily statistically significant because of the lower degradation.

When the co-suspensions of SS and MG were initially treated by method I, methanol was used as the washing solvent. The HPLC chromatograms at that time showed two degradation peaks, where one was identified as simvastatin acid with a shorter retention time and the other was observed with a longer retention time (Supplemental Fig. 10). The latter peak was estimated to be due to a simvastatin acid methyl ester, as a result of the NMR spectroscopic and mass spectrometric (accurate mass) analyses of the HPLC fraction corresponding to the peak (data are not shown). Yang et al. [25] have also reported that simvastatin acid methyl ester was formed from simvastatin acid in an alkaline aqueous methanolic solution. These findings indicated that the degradation due to the SSM itself could be overestimated by the treatment with methanol. Thus, the SS suspensions were treated with acetonitrile, by which simvastatin acid was observed as the sole degradation product in the co-suspensions with MG.

In general, statins are known to show a pH-dependent interconversion between the active hydroxy acid form and the lactone one [24, 25, 34, 35]. It has been reported that statins exist as their mixture under strong acidic conditions, whereas under alkaline conditions they exclusively exist as hydroxy acid forms. Simvastatin is used as a lactone form, *i.e.*, a prodrug, and it exerts pharmacological effects after being hydrolyzed to the active hydroxy acid form in the body. This study revealed that simvastatin is partially transformed into a less lipophilic hydroxy acid, simvastatin acid, under the condition of the SSM with MG. Such a transformation before ingestion possibly decreases the efficacy of simvastatin since simvastatin acid is poorly absorbed into the gastrointestinal tract when compared with simvastatin.

It has also been reported that ezetimibe undergoes the cleavage of the β-lactam and the ring transformation into the pyran compound under alkaline conditions [28]. The reactions are dependent on the pH of the solution, where the pyran compound is mainly formed at pH 7 to 12.5. Although the decomposition of ezetimibe into the pyran compound has been demonstrated to occur under the strong conditions in the literature, the same decomposition was found to slightly occur also under the condition of the SSM with MG.

The temperature (55°C) of the water used for the SSM is a rough indication. The temperature was selected because it was maintained at about 37°C after having been left for 10 min at room temperature [8]. In general, the temperature of the water used is not measured in clinical settings, and thus the co-suspensions are possibly prepared at higher temperatures. The degradation can be accelerated in such cases. The degradation observed in this study seems to also occur in the co-suspension with other alkaline drugs such as magnesium hydroxide [14].

This study dealt with the specific generic drugs of CLDs, and it is of interest whether similar results are obtained for other formulations including the original and generic drugs. Although various acidic and basic additives are used, the amounts are considered smaller than the co-suspended MgO (330 mg) in most cases, because relatively small tablets (less than 200 mg) are generally used except some orally disintegrating tablets. Thus, the alkalinity and putative interactions are considered to be largely dependent on MgO. It can therefore be presumed that comparable results with the present ones will also be obtained using the amount of MgO for most of other formulations.

## Conclusions

In this paper, the chemical stability of typical CLDs in a co-suspension with a specific amount of MgO was investigated under an SSM condition. We found that atorvastatin and pravastatin can be subjected to the co-suspension without significant chemical changes. However, it is preferable that the co-suspension of rosuvastatin is rapidly prepared and used after having been disintegrated for 10 min. For simvastatin and ezetimibe, the co-suspension with MgO should be avoided because of their partial degradation. Although this study was performed for specific generic drugs, the data should be considered informative for the co-suspensions of other formulations with MgO.

### Supplementary Information

**Additional file 1: Supplemental Fig. 1.** ^1^H-decoupled ^13^C NMR spectrum of the chloroform extract from the co-suspension of SS ad MG. solvent, CDCl_3_.

**Additional file 2: Supplemental Fig. 2.** Correlation spectroscopic analysis of the chloroform extract from the co-suspension of SS and MG. solvent, CDCl_3_.

**Additional file 3: Supplemental Fig. 3.** Heteronuclear multiple quantum correlation spectrum of the chloroform extract from the co-suspension of SS and MG. solvent, CDCl_3_.

**Additional file 4: Supplemental Fig. 4.** Heteronuclear multiple bond coherence spectrum of the chloroform extract from the co-suspension of SS and MG. solvent, CDCl_3_.

**Additional file 5: Supplemental Fig. 5.** ^1^H NMR spectra of ezetimibe (A) and its degradation product isolated from the co-suspension of EZ and MG (B).

**Additional file 6: Supplemental Fig. 6.** ^1^H-decoupled ^13^C NMR spectrum of the pyran compound. solvent, DMSO-*d*_6_.

**Additional file 7: Supplemental Fig. 7.** Correlation spectroscopic analysis of the pyran compound. solvent, DMSO-*d*_6_.

**Additional file 8: Supplemental Fig. 8.** Heteronuclear multiple quantum correlation spectrum of the pyran compound. solvent, DMSO-*d*_6_.

**Additional file 9: Supplemental Fig. 9.** Heteronuclear multiple bond coherence spectrum of the pyran compound. solvent, DMSO-*d*_6_.

**Additional file 10: Supplemental Table 1.** Intra- and inter-day precision and accuracy^a^.

**Additional file 11: Supplemental Table 2.** Calibration curves, LOD, and LOQ.

**Additional file 12: Supplemental Fig. 10.** HPLC chromatogram of aqueous methanolic extract from the co-suspension of SS and MG.

## Data Availability

The datasets used and/or analyzed during the current study are available from the corresponding author on reasonable request.

## Abbreviations

AS: Atorvastatin tablets 5 mg
CLD: Cholesterol-lowering drug
DMSO-*d*_6_: Deuterated dimethyl sulfoxide
ESI–MS: Electrospray ionization mass spectrum
EZ: Ezetimibe tablets 10 mg
HPLC: High-performance liquid chromatography
LOD: Limit of detection
LOQ: Limit of quantitation
MG: Magmitt tablets 330 mg
NMR: Nuclear magnetic resonance
PS: Pravastatin sodium tablets 5 mg
RS: Rosuvastatin tablets 2.5 mg
SS: Simvastatin tablets 5 mg
SSM: Simple suspension method

## References

[1] Christmas C, Rogus-Pulia N (2019). Swallowing disorders in the older population. J Am Geriatr Soc.

[2] Sestili M, Logrippo S, Cespi M (2018). Potentially inappropriate prescribing of oral solid medications in elderly dysphagic patients. Pharmaceutics.

[3] Williams NT (2008). Medication administration through enteral feeding tubes. Am J Health-Syst Pharm.

[4] Lau ETL, Steadman KJ, Cichero JAY, Nissen LM (2018). Dosage form modification and oral drug delivery in older people. Adv Drug Deliv Rev.

[5] Thong MY, Manrique YJ, Steadman KJ (2018). Drug loss while crushing tablets: Comparison of 24 tablet crushing devices. PLoS One.

[6] Crul M, Breukels O, Ng S, Le Feber M, Kuijpers E, Smeets O (2023). Limited health risks in performing drug reconstitution and handling tasks in pharmacies - results of an occupational risk assessment study. J Occup Environ Med.

[7] Murahashi T, Arai M, Ogata K, Matsumoto M, Higuchi T (2022). Occupational exposure of pharmacists to drugs during tablet crushing and its countermeasures. Fundam Toxicol Sci.

[8] Kunieda K, Kurata N, Yoshimatsu Y, Ohno T, Shigematsu T, Fujishima I (2022). A safe way to administer drugs through a nutrition tube-the simple suspension method. Dysphagia.

[9] Tanaka R, Eto D, Goto K (2021). Pharmacokinetic and adsorptive analyses of administration of oral voriconazole suspension *via* enteral feeding tube in intensive care unit patients. Biol Pharm Bull.

[10] Morita TO, Yamaguchi A, Kimura S (2016). Stability of lenalidomide suspension after preparation by a simple suspension method for enteral tube administration. J Oncol Pharm Pract.

[11] Masaoka Y, Kawasaki Y, Kikuoka R (2021). Development of an appropriate simple suspension method for valganciclovir medication. J Pharm Health Care Sci.

[12] Mori H, Tack J, Suzuki H (2021). Magnesium oxide in constipation. Nutrients.

[13] Kang SJ, Cho YS, Lee TH (2021). Medical management of constipation in elderly patients: systematic review. J Neurogastroenterol Motil.

[14] Ogawa R, Echizen H (2011). Clinically significant drug interactions with antacids: an update. Drugs.

[15] Aoki M, Naya M, Arima S (2021). Mixture of clopidogrel bisulfate and magnesium oxide tablets reduces clopidogrel dose administered through a feeding tube. J Pharm Health Care Sci.

[16] Suryani N, Sugiyama E, Kurata N, Sato H (2013). Stability of ester prodrugs with magnesium oxide using the simple suspension method. Jpn J Pharm Health Care Sci.

[17] Rossi M, Fabris E, Barbisan D, Massa L, Sinagra G (2022). Lipid-lowering drug therapy: critical approach for implementation in clinical practice. Am J Cardiovasc Drugs.

[18] Muscoli S, Ifrim M, Russo M (2022). Current options and future perspectives in the treatment of dyslipidemia. J Clin Med.

[19] Khedr A (2007). Stability-indicating high-performance liquid chromatographic assay of atorvastatin with fluorescence detection. J AOAC Int.

[20] Chaudhari BG, Patel NM, Shah PB (2007). Stability indicating RP-HPLC method for simultaneous determination of atorvastatin and amlodipine from their combination drug products. Chem Pharm Bull.

[21] Önal A, Sagirli O (2006). Development of a selective LC method for the determination of pravastatin sodium. Chromatographia.

[22] Machairas G, Panderi I, Geballa-Koukoula A (2018). Development and validation of a hydrophilic interaction liquid chromatography method for the quantitation of impurities in fixed-dose combination tablets containing rosuvastatin and metformin. Talanta.

[23] Belal F, Ibrahim F, Khedr A, Elawady T (2014). Stability indicating TLC method for the determination of rosuvastatin and identification of some degradation products using electrospray ionization mass spectrometry. J Liq Chromatogr Relat Technol.

[24] Alvarez-Lueje A, Valenzuela C, Squella JA, Núñez-Vergara LJ (2005). Stability study of simvastatin under hydrolytic conditions assessed by liquid chromatography. J AOAC Int.

[25] Yang DJ, Hwang LS (2006). Study on the conversion of three natural statins from lactone forms to their corresponding hydroxy acid forms and their determination in Pu-Erh tea. J Chromatogr A.

[26] Goel A, Baboota S, Sahni JK (2013). Development and validation of stability-indicating assay method by UPLC for a fixed dose combination of atorvastatin and ezetimibe. J Chromatogr Sci.

[27] Sánta Z, Kóti J, Szőke K, Vukics K, Szántay C (2012). Structure of the major degradant of ezetimibe. J Pharm Biomed Anal.

[28] Baťová J, Imramovský A, HájÍček J, Hejtmánková L, Hanusek J (2014). Kinetics and mechanism of the base-catalyzed rearrangement and hydrolysis of ezetimibe. J Pharm Sci.

[29] Kato G, Mitome H, Teshima K (2023). Study on the use of ozone water as a chemical decontamination agent for antineoplastic drugs in clinical settings. Ann Work Expo Health.

[30] Kublin E, Malanowicz E, Kaczmarska-Graczyk B, Czerwińska K, Wyszomirska E, Mazurek AP (2015). Development of chromatographic method for determination of drugs reducing cholesterol level-statins and ezetimibe. Acta Pol Pharm.

[31] Takano Y, Kabe H, Mizoi K (2021). Effect of magnesium oxide on the stability of ACE inhibitors in simple suspension method. Jpn J Pharm Health Care Sci.

[32] Russell TL, Berardi RR, Barnett JL (1993). Upper gastrointestinal pH in seventy-nine healthy, elderly, North American men and women. Pharm Res.

[33] Martin PD, Schneck DW, Dane AL, Warwick MJ (2008). The effect of a combination antacid preparation containing aluminium hydroxide and magnesium hydroxide on rosuvastatin pharmacokinetics. Curr Med Res Opin.

[34] Geboers S, Stappaerts J, Tack J, Annaert P, Augustijns P (2016). In vitro and in vivo investigation of the gastrointestinal behavior of simvastatin. Int J Pharm.

[35] Hoffmann M, Nowosielski M (2008). DFT study on hydroxy acid-lactone interconversion of statins: the case of atorvastatin. Org Biomol Chem.

