# Supplementary material for: A study on the chemical stability of cholesterol-lowering drugs in concomitant simple suspensions with magnesium oxide
Source: J Pharm Health Care Sci. 2023 Aug 29;9:32. doi: 10.1186/s40780-023-00301-1 (PMC10464426; doi:10.1186/s40780-023-00301-1)
Supplement: Supplementary file 1 — Additional file 1: Supplemental Fig. 1. 1H-decoupled 13C NMR spectrum of the chloroform extract from the co-suspension of SS ad MG. solvent, CDCl3. [file 40780_2023_301_MOESM1_ESM.pdf]

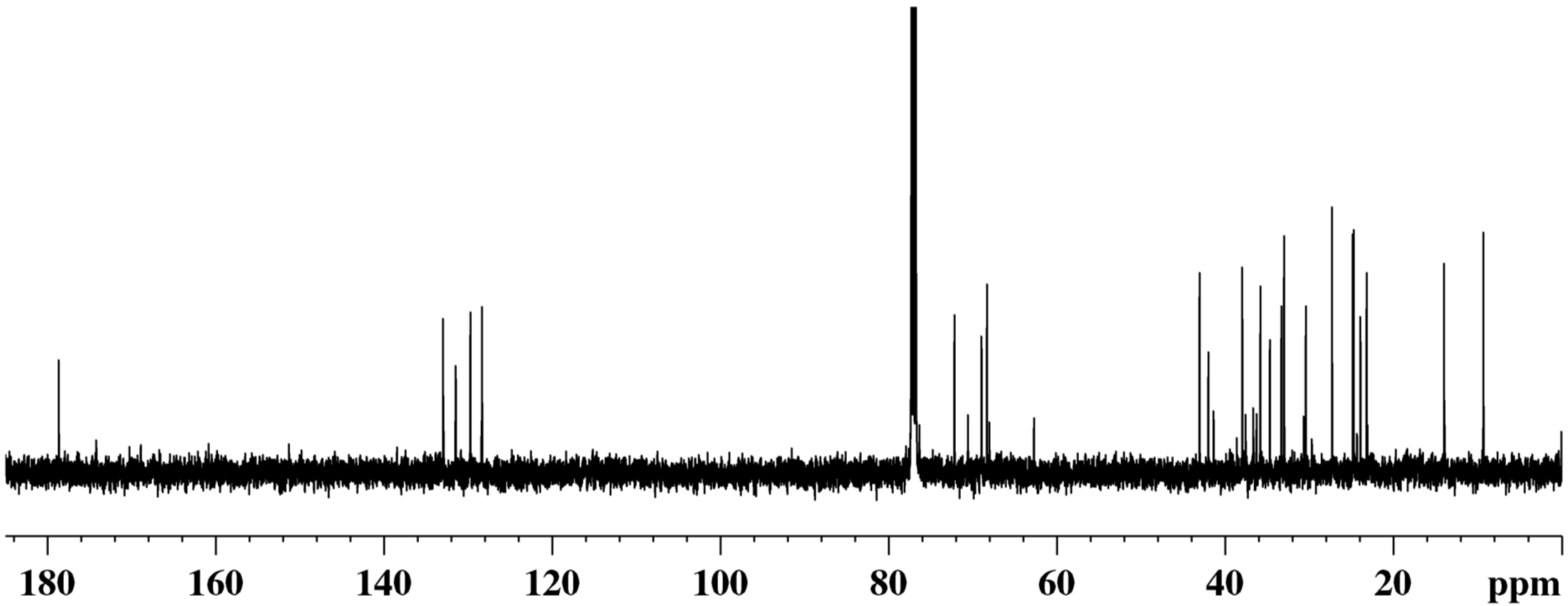


Supplemental Fig. 1 $^{1}H$-decoupled $^{13}C$ NMR spectrum of the chloroform extract from the co-suspension of SS and MG. solvent, $CDCl_3$.
