# Supplementary material for: A study on the chemical stability of cholesterol-lowering drugs in concomitant simple suspensions with magnesium oxide
Source: J Pharm Health Care Sci. 2023 Aug 29;9:32. doi: 10.1186/s40780-023-00301-1 (PMC10464426; doi:10.1186/s40780-023-00301-1)
Supplement: Supplementary file 2 — Additional file 2: Supplemental Fig. 2. Correlation spectroscopic analysis of the chloroform extract from the co-suspension of SS and MG. solvent, CDCl3. [file 40780_2023_301_MOESM2_ESM.pdf]

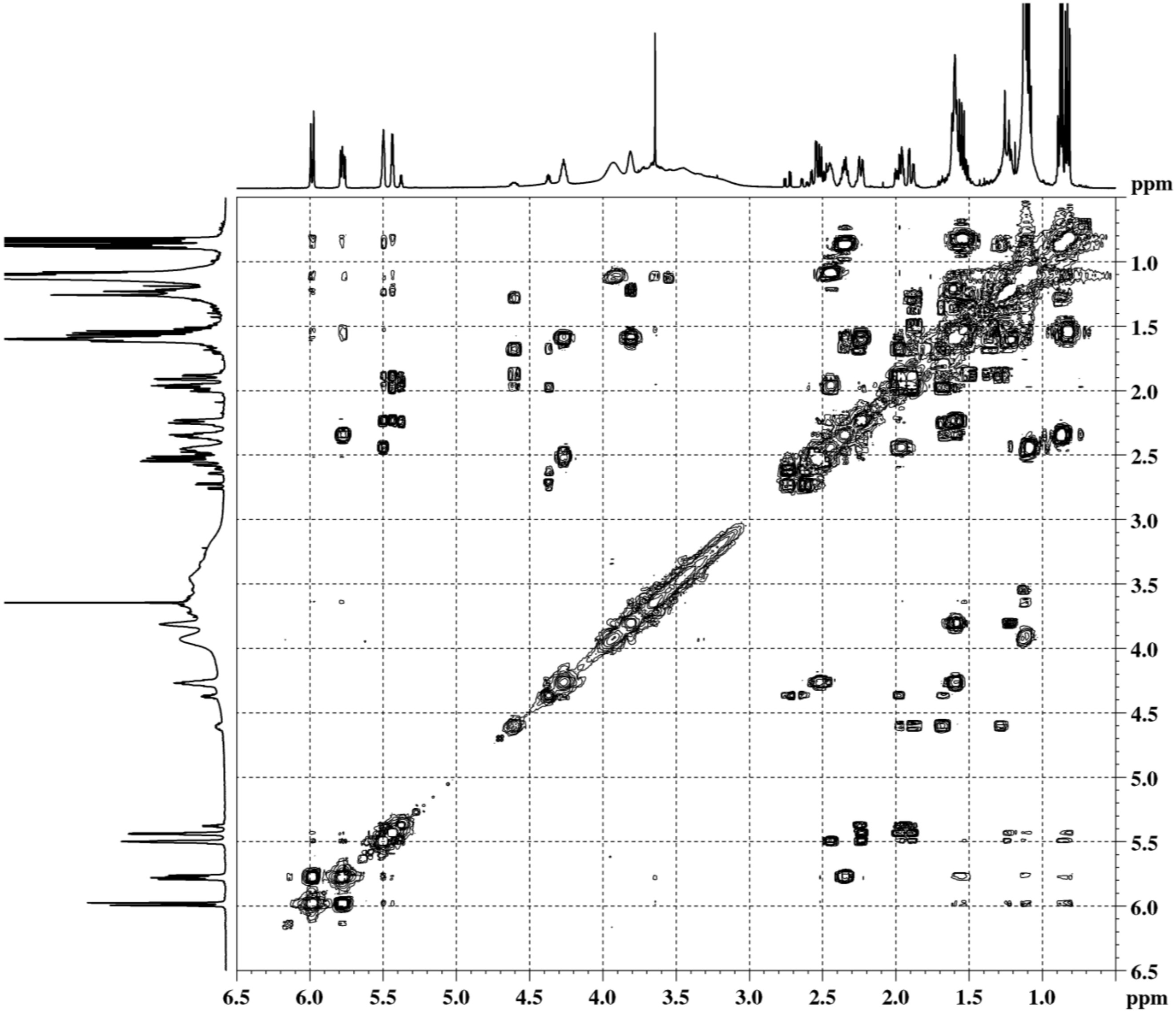

Supplemental Fig. 2 Correlation spectroscopic analysis of the chloroform extract from the co-suspension of SS and MG. solvent, $CDCl_3$.
