# Supplementary material for: A study on the chemical stability of cholesterol-lowering drugs in concomitant simple suspensions with magnesium oxide
Source: J Pharm Health Care Sci. 2023 Aug 29;9:32. doi: 10.1186/s40780-023-00301-1 (PMC10464426; doi:10.1186/s40780-023-00301-1)
Supplement: Supplementary file 5 — Additional file 5: Supplemental Fig. 5. 1H NMR spectra of ezetimibe (A) and its degradation product isolated from the co-suspension of EZ and MG (B). [file 40780_2023_301_MOESM5_ESM.pdf]

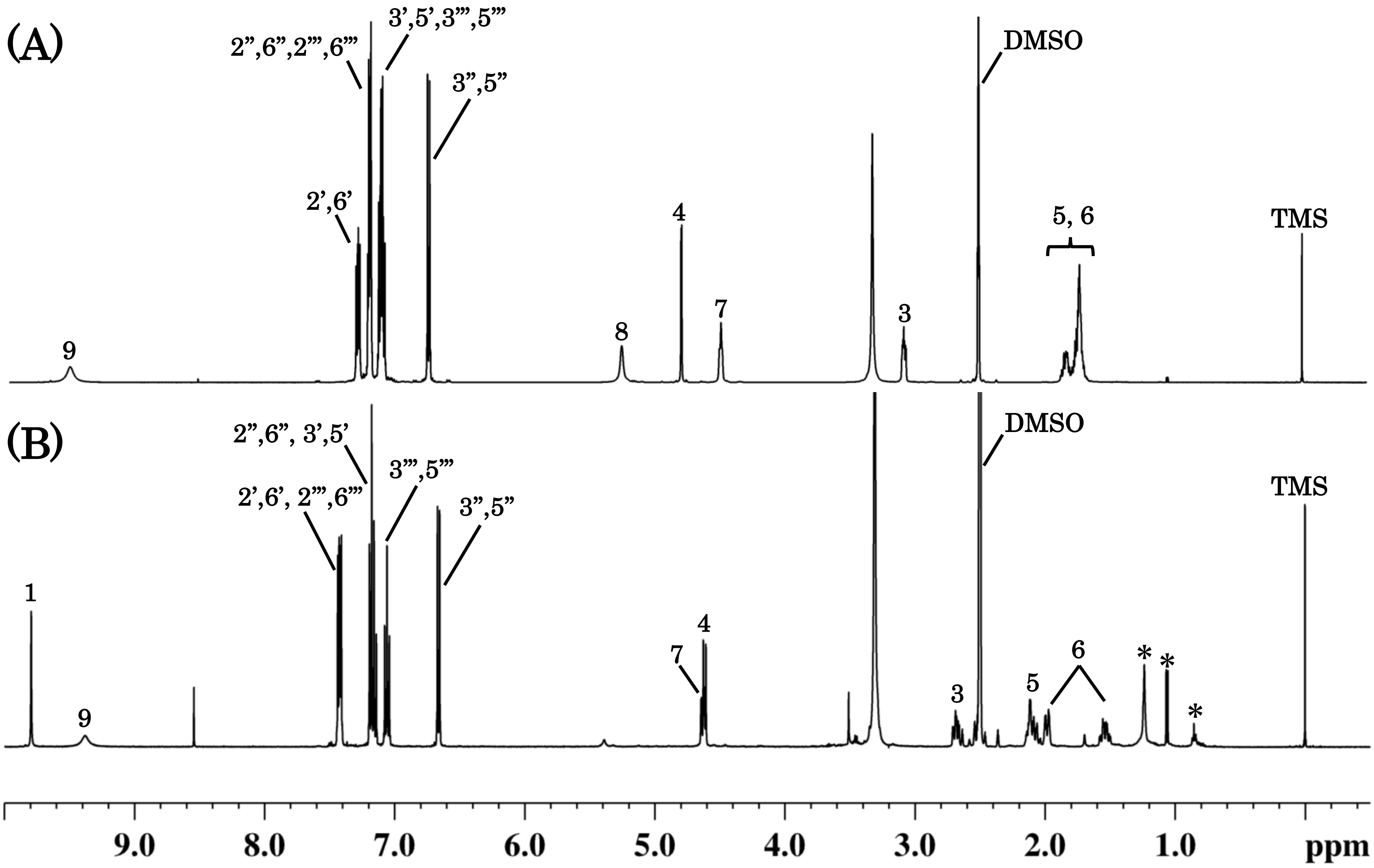

Supplemental Fig. 5 ^{1}H NMR spectra of ezetimibe (A) and its degradation product isolated from the co-suspension of EZ and MG (B).

The spectra were measured in DMSO-d_6. The numeric characters on the spectra (A) and (B) show assignments of the signals to the positions of the chemical structures shown in Fig. 1 (ezetimibe) and Fig. 4 (the degradation product, a pyran compound), respectively. *, impurities.
