# Supplementary figures and images for: A study on the chemical stability of cholesterol-lowering drugs in concomitant simple suspensions with magnesium oxide
Source: J Pharm Health Care Sci. 2023 Aug 29;9:32. doi: 10.1186/s40780-023-00301-1 (PMC10464426; doi:10.1186/s40780-023-00301-1)

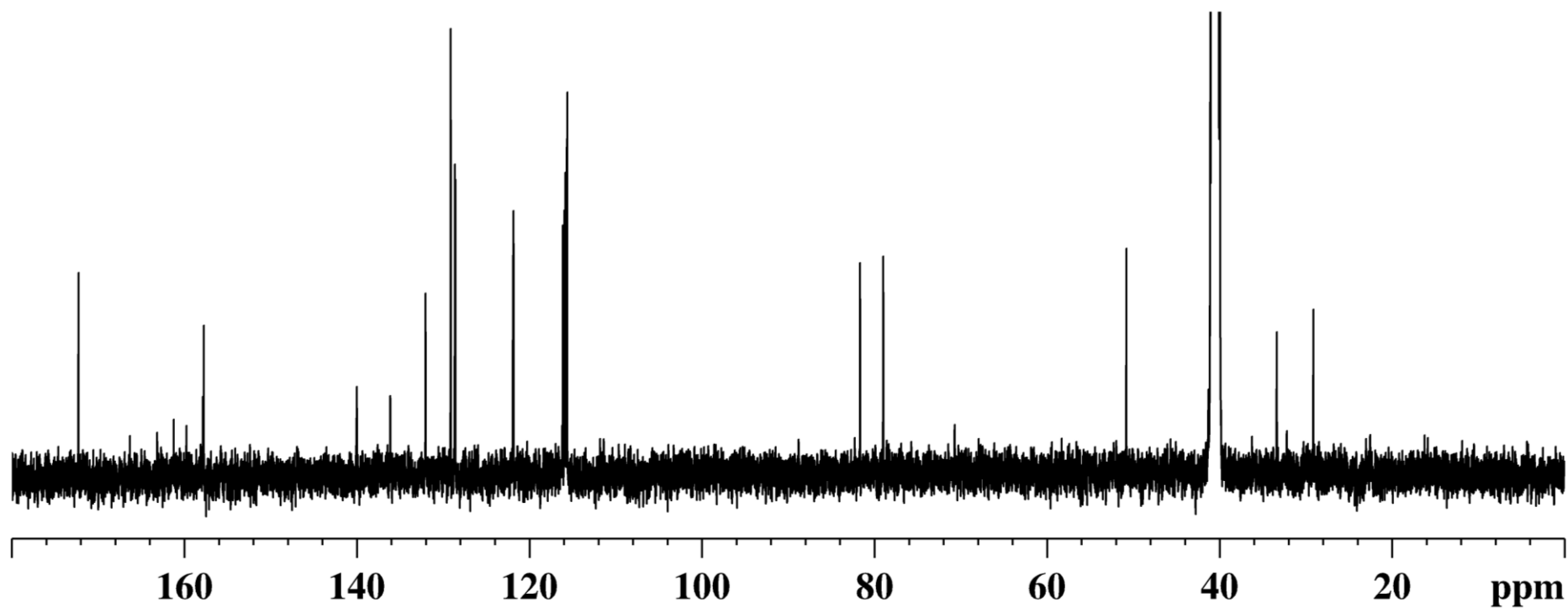

Supplemental Fig. 6

$^1\text{H}$ -decoupled  $^{13}\text{C}$  NMR spectrum of the pyran compound.  
solvent,  $\text{DMSO}-d_6$ .

Supplement: Supplementary file 6 — Additional file 6: Supplemental Fig. 6. 1H-decoupled 13C NMR spectrum of the pyran compound. solvent, DMSO-d6. [file 40780_2023_301_MOESM6_ESM.pdf]

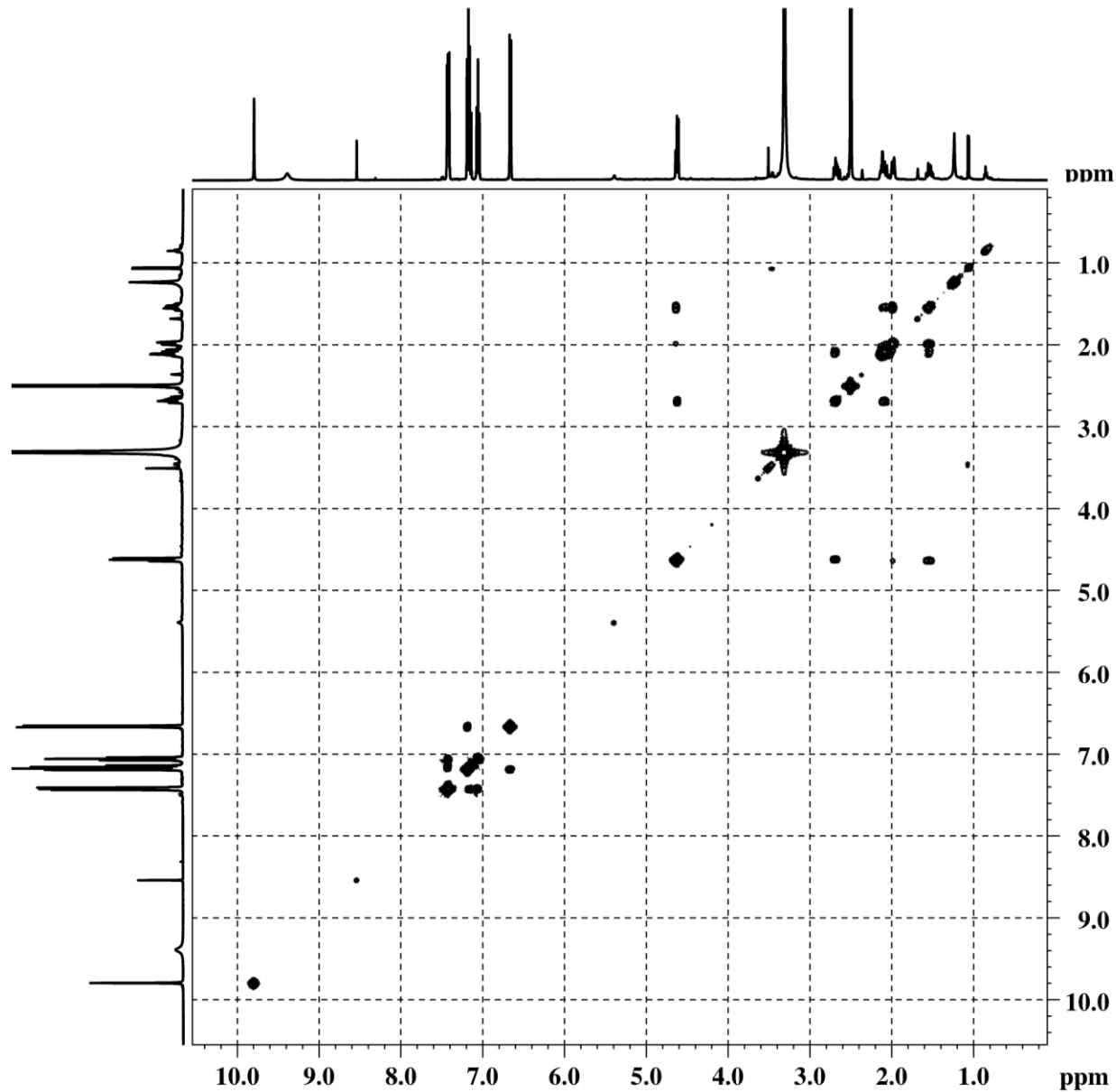

Supplemental Fig. 7     Correlation spectroscopic analysis of the pyran compound.  
solvent, DMSO- $d_6$ .

Supplement: Supplementary file 7 — Additional file 7: Supplemental Fig. 7. Correlation spectroscopic analysis of the pyran compound. solvent, DMSO-d6. [file 40780_2023_301_MOESM7_ESM.pdf]
