# Supplementary material for: A study on the chemical stability of cholesterol-lowering drugs in concomitant simple suspensions with magnesium oxide
Source: J Pharm Health Care Sci. 2023 Aug 29;9:32. doi: 10.1186/s40780-023-00301-1 (PMC10464426; doi:10.1186/s40780-023-00301-1)
Supplement: Supplementary file 10 — Additional file 10: Supplemental Table 1. Intra- and inter-day precision and accuracya. [file 40780_2023_301_MOESM10_ESM.docx]

Supplemental Table 1. Intra- and inter-day precision and accuracy^a^

| Drug | Concentration (μg/mL) |  | Intra-day | | | |  | Inter-day | | | |
| --- | --- | --- | --- | --- | --- | --- | --- | --- | --- | --- | --- |
|  |  |  | Mean (μg/mL) | Standard deviation | Relative standard deviation (%) | Bias (%) |  | Mean (μg/mL) | Standard deviation | Relative standard deviation (%) | Bias (%) |
| atorvastatin | 0.50 |  | 0.50 | 0.0055 | 1.1 | 1.9 |  | 0.51 | 0.0063 | 1.2 | 2.9 |
|  | 1.0 |  | 0.97 | 0.012 | 1.2 | −3.1 |  | 0.97 | 0.0061 | 0.62 | −2.4 |
|  | 3.0 |  | 3.0 | 0.051 | 1.7 | −0.22 |  | 3.0 | 0.024 | 0.81 | −0.23 |
| pravastatin | 0.47 |  | 0.49 | 0.0063 | 1.3 | 2.9 |  | 0.48 | 0.0079 | 1.6 | 1.7 |
|  | 0.95 |  | 0.98 | 0.0091 | 0.92 | 3.7 |  | 0.98 | 0.0052 | 0.53 | 3.2 |
|  | 2.8 |  | 3.0 | 0.028 | 0.94 | 4.5 |  | 3.0 | 0.048 | 1.6 | 4.3 |
| rosuvastatin | 0.30 |  | 0.31 | 0.0059 | 1.9 | 3.9 |  | 0.31 | 0.0048 | 1.6 | 2.8 |
|  | 0.50 |  | 0.51 | 0.0062 | 1.2 | 1.8 |  | 0.51 | 0.012 | 2.4 | 2.1 |
|  | 1.5 |  | 1.4 | 0.021 | 1.5 | −3.9 |  | 1.5 | 0.034 | 2.4 | −2.9 |
| simvastatin | 0.30 |  | 0.30 | 0.0051 | 1.7 | 1.4 |  | 0.30 | 0.0070 | 2.3 | −0.76 |
|  | 1.0 |  | 0.99 | 0.0049 | 0.50 | −1.5 |  | 0.97 | 0.018 | 1.9 | −3.5 |
|  | 3.0 |  | 2.9 | 0.0075 | 0.26 | −2.6 |  | 2.9 | 0.039 | 1.4 | −4.1 |
| ezetimibe | 0.96 |  | 0.92 | 0.0018 | 0.19 | −4.0 |  | 0.92 | 0.0061 | 0.67 | −4.2 |
|  | 1.9 |  | 1.9 | 0.024 | 1.3 | −2.9 |  | 1.9 | 0.0016 | 0.088 | −2.8 |
|  | 5.8 |  | 5.7 | 0.031 | 0.54 | −1.1 |  | 5.7 | 0.0047 | 0.082 | −1.2 |

^a^The quality control samples were analyzed five time using the HPLC system for the intra-day precision, and such experiments were performed on three different days for the inter-day precision.
