# Supplementary material for: A study on the chemical stability of cholesterol-lowering drugs in concomitant simple suspensions with magnesium oxide
Source: J Pharm Health Care Sci. 2023 Aug 29;9:32. doi: 10.1186/s40780-023-00301-1 (PMC10464426; doi:10.1186/s40780-023-00301-1)
Supplement: Supplementary file 11 — Additional file 11: Supplemental Table 2. Calibration curves, LOD, and LOQ. [file 40780_2023_301_MOESM11_ESM.docx]

Supplemental Table 2. Calibration curves, LOD, and LOQ

| Drug | Slope | Intercept | *r* ^2^ | Concentration range (μg/mL) | LOD (ng/mL) | LOQ  (ng/mL) |
| --- | --- | --- | --- | --- | --- | --- |
| atorvastatin | 24071 | −710.08 | 0.9998 | 0.25−3.96 | 16 | 47 |
| pravastatin | 25765 | +517.02 | 0.9998 | 0.31−4.18 | 65 | 262 |
| rosuvastatin | 23669 | +584.67 | 0.9999 | 0.24−1.95 | 49 | 210 |
| simvastatin | 29927 | −8.2361 | 0.9999 | 0.24−3.92 | 37 | 157 |
| ezetimibe | 22969 | −1890.3 | 0.9996 | 0.52−8.33 | 36 | 143 |
