# Supplementary material for: A study on the chemical stability of cholesterol-lowering drugs in concomitant simple suspensions with magnesium oxide
Source: J Pharm Health Care Sci. 2023 Aug 29;9:32. doi: 10.1186/s40780-023-00301-1 (PMC10464426; doi:10.1186/s40780-023-00301-1)
Supplement: Supplementary file 12 — Additional file 12: Supplemental Fig. 10. HPLC chromatogram of aqueous methanolic extract from the co-suspension of SS and MG. [file 40780_2023_301_MOESM12_ESM.pdf]

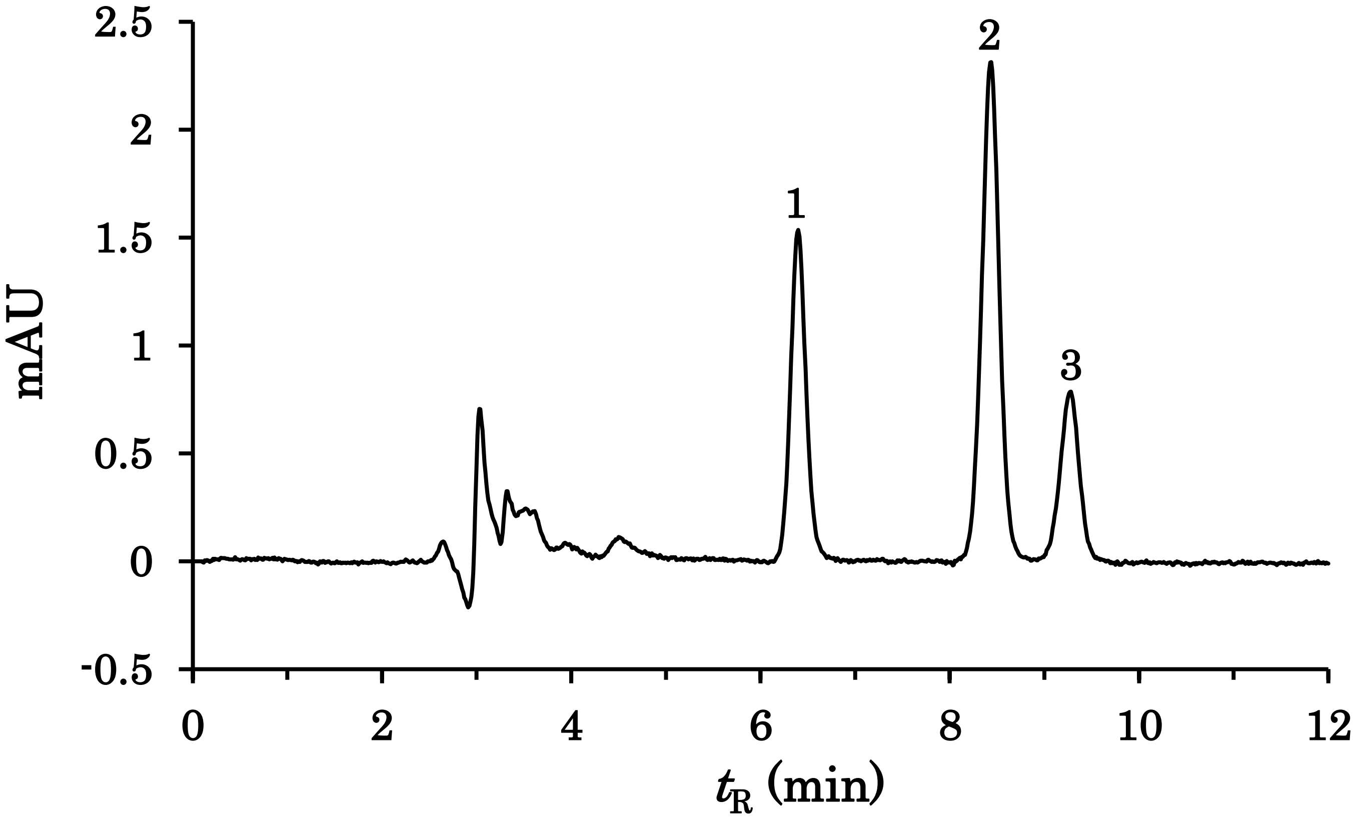


Supplemental Fig. 10 HPLC chromatogram of aqueous methanolic extract from the co-suspension of SS and MG.

symbols: 1, simvastatin acid; 2, simvastatin; 3, simvastatin acid methyl ester.
